# How the Brain Decides When to Work and When to Rest: Dissociation of Implicit-Reactive from Explicit-Predictive Computational Processes

**DOI:** 10.1371/journal.pcbi.1003584

**Published:** 2014-04-17

**Authors:** Florent Meyniel, Lou Safra, Mathias Pessiglione

**Affiliations:** Motivation, Brain & Behavior (MBB) team, Institut du Cerveau et de la Moelle épinière (ICM), Groupe Hospitalier Pitié-Salpêtrière, Université Pierre et Marie Curie (UPMC – Paris 6), Paris, France; Oxford University, United Kingdom

## Abstract

A pervasive case of cost-benefit problem is how to allocate effort over time, i.e. deciding when to work and when to rest. An economic decision perspective would suggest that duration of effort is determined beforehand, depending on expected costs and benefits. However, the literature on exercise performance emphasizes that decisions are made on the fly, depending on physiological variables. Here, we propose and validate a general model of effort allocation that integrates these two views. In this model, a single variable, termed cost evidence, accumulates during effort and dissipates during rest, triggering effort cessation and resumption when reaching bounds. We assumed that such a basic mechanism could explain implicit adaptation, whereas the latent parameters (slopes and bounds) could be amenable to explicit anticipation. A series of behavioral experiments manipulating effort duration and difficulty was conducted in a total of 121 healthy humans to dissociate implicit-reactive from explicit-predictive computations. Results show 1) that effort and rest durations are adapted on the fly to variations in cost-evidence level, 2) that the cost-evidence fluctuations driving the behavior do not match explicit ratings of exhaustion, and 3) that actual difficulty impacts effort duration whereas expected difficulty impacts rest duration. Taken together, our findings suggest that cost evidence is implicitly monitored online, with an accumulation rate proportional to actual task difficulty. In contrast, cost-evidence bounds and dissipation rate might be adjusted in anticipation, depending on explicit task difficulty.

## Author Summary

Imagine that ahead of you is a long time of work: when will you take a break? This sort of issue – how to allocate effort over time – has been addressed by distinct theoretical fields, with different emphasis on reactive and predictive processes. An intuitive view is that you start working, stop when you are tired, and start again when fatigue goes away. Biologically, this means that decisions are taken when some physiological variable reaches a given bound on the risk of homeostatic failure. In a more economic perspective, fatigue translates into effort cost, which must be anticipated and compared to expected benefit before engaging an action. We proposed a computational model that bridges these perspectives from sport physiology and decision theory. Decisions are made in reaction to bounds being reached by an implicit cost variable that accumulates during effort, at a rate proportional to task difficulty, and dissipates during rest. However, some latent parameters (bounds and dissipation rate) are adjusted in anticipation, depending on explicit costs and benefits. This model was supported by behavioral data obtained using a paradigm where participants squeeze a handgrip to win a monetary payoff proportional to effort duration.

## Introduction

Suppose that you are given a job whose payoff is proportional to the effort made within a limited time, say for instance the number of Christmas cards sold at the end of the day. Maximizing your payoff would require running from house to house, but this effort would induce such fatigue that you decide to walk from time to time. This sort of situation can be examined through economic decision theory, which would suggest you to write down the expected costs and benefits, and try to figure out whether the effort is worthy. If the cost of a given effort is anticipated to increase with fatigue [1], [2], then you will find an optimal duration that can be determined before engaging any action. Yet the literature on exercise performance has developed a different perspective on this issue [3], [4], which would suggest that you start by running, and only stop when some physiological variable, for instance in cardiovascular function (such as heart beat rate) or in muscular metabolism (such as lactate concentration), attains a given limit [5], [6]. In other words, effort cessation would be a reaction to homeostatic failure, and would not require any explicit anticipation of effort cost.

**Figure 1:**
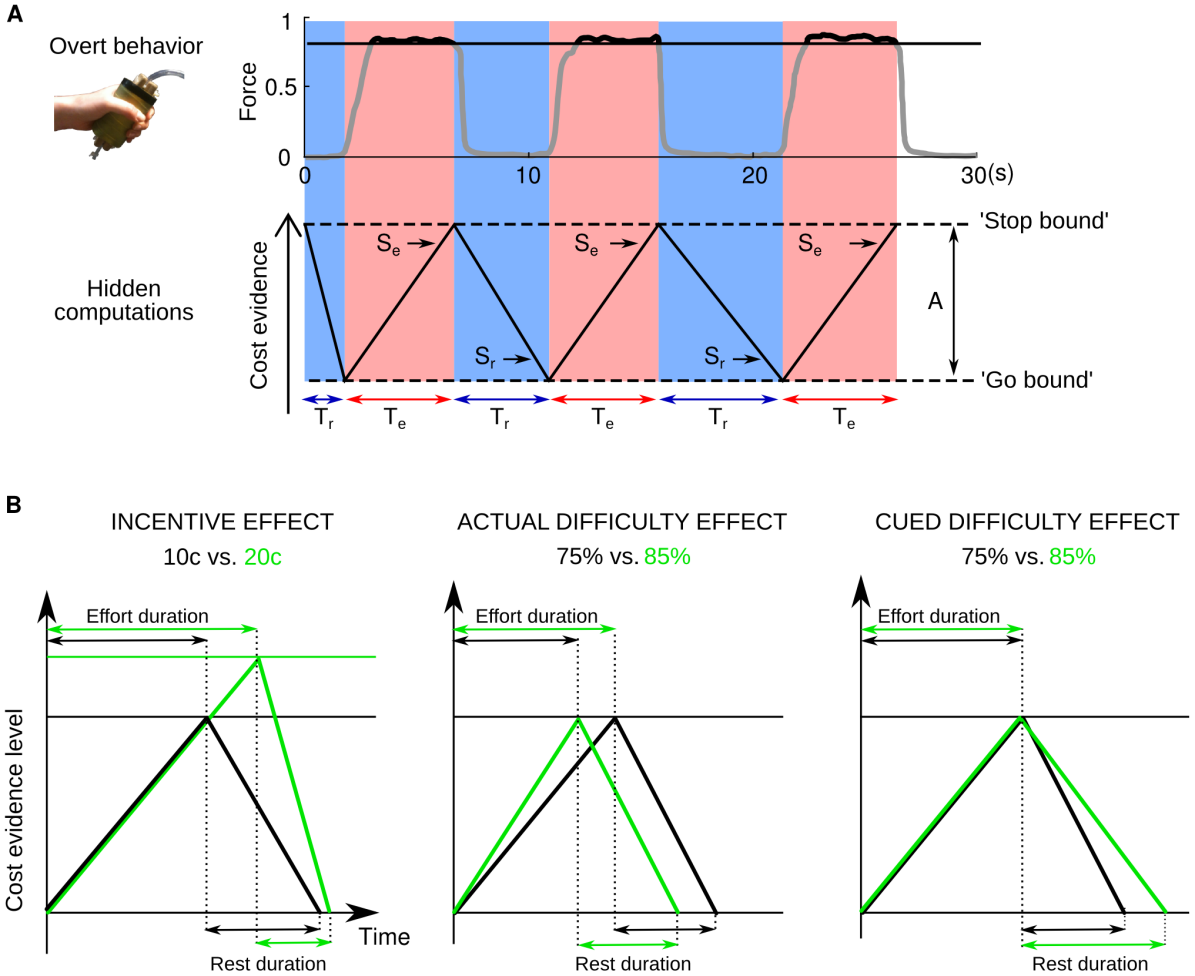
Computational model of effort allocation over time. A. Application of the accumulation-to-bound principle to cost-evidence monitoring. The graph displays an example trial from the Effort Allocation Task, with the observed force time series on top of the theoretical cost evidence. Force level was normalized by the participant's maximal force (estimated prior to the experiment). It is traced in black (not gray) when the effort is rewarded, i.e. when above the target force level (80% of maximal force in this example). The three effort periods shown in red were defined using both the force level and its temporal derivative. The modeled cost evidence accumulates during effort, with slope Se, to an upper bound triggering effort cessation, and then dissipates during rest with slope Sr, to a lower bound triggering effort resumption. The amplitude of cost-evidence fluctuations is denoted A. The accumulation model was built to fit two dependent measures: effort duration (Te) and rest duration (Tr). B. Illustration of how the experimental factors (monetary incentive, actual difficulty, expected difficulty) affect cost-evidence monitoring. Bayesian model comparison dissociated the computational effects of experimental factors: higher incentives increase the amplitude between bounds and the dissipation rate; higher actual difficulty steepens the accumulation rate; higher expected difficulty shallows the dissipation rate. Mathematical equations of the winning model are given in the results section.

These two extreme perspectives have obvious limitations. The physiological view does not account for the effect of expectations that might pre-configure behavioral performance [4], [7], [8]. The economic view does not integrate the constraints imposed by physiological reactions, which might be difficult to anticipate [9]. Here, we intend to overcome these limitations by integrating the two perspectives into the same computational model. Furthermore, we have built this model so as to explain the duration not only of effort exertion but also of rest (recovery from fatigue). Let us assume that a single waning and waxing variable triggers decisions to stop and restart effort exertion when reaching bounds (see Figure 1A for a graphical presentation). As this variable linearly accumulates during effort and dissipates at rest, it can be seen as a simple reflection of physiological reactions that predict the proximity of homeostatic failure. Alternatively, it can be interpreted as tracking cost increase with fatigue, by integrating past effort over time. Thus, the basic architecture of the model (the accumulation-to-bound principle) can account for implicit, online adaptation to actual effort costs, complying with physiological constraints. On this basis, the modulation of the model latent parameters (slopes and bounds) could allow for anticipatory adjustments, depending on explicit costs and benefits (see Figure 1B for a graphical illustration).

**Figure 2:**
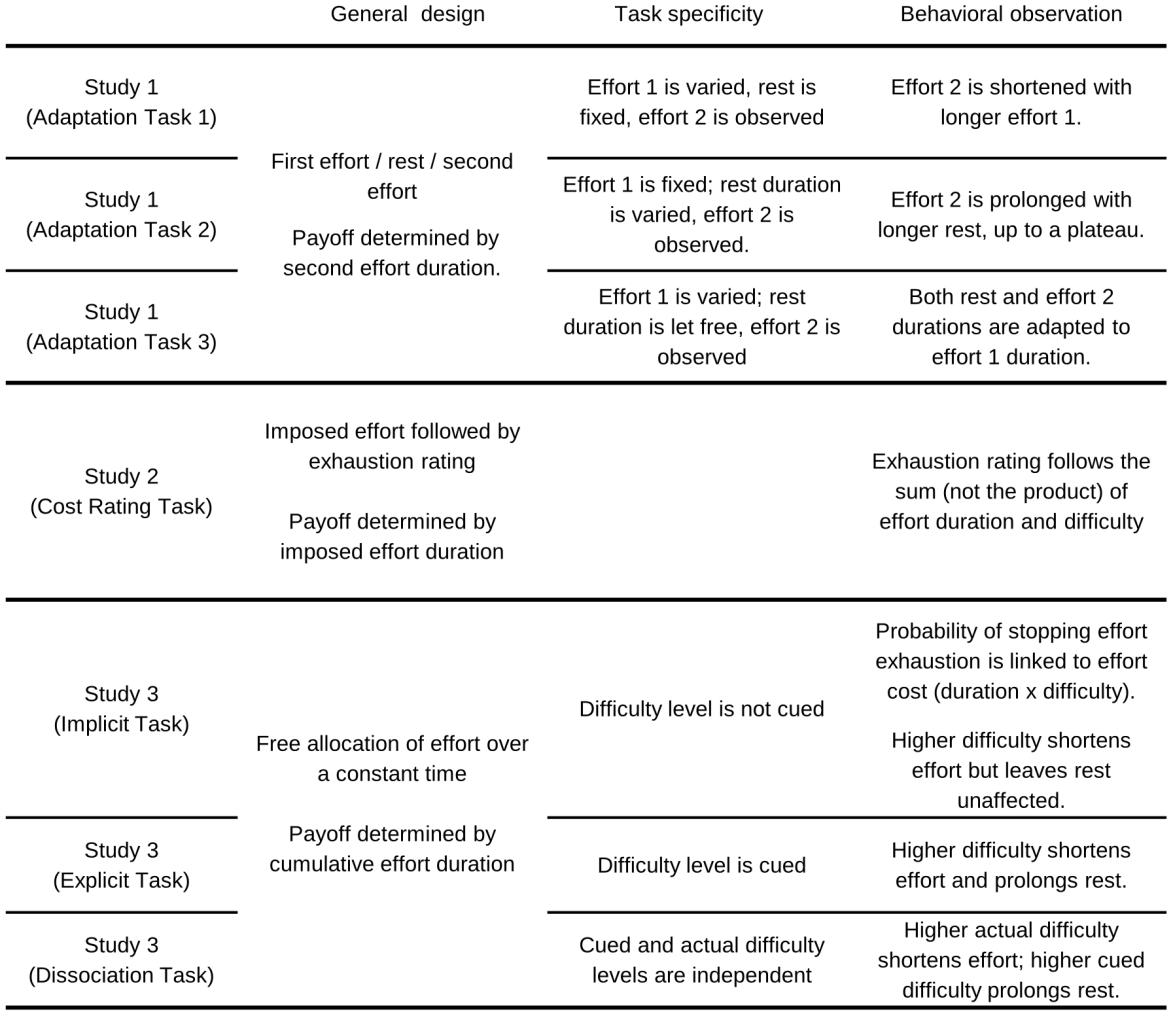
Summary of experimental manipulations and behavioral findings.

To dissociate the effects of actual and expected effort costs, we developed seven variants of a paradigm that was employed in a previous paper [10] to identify the neural underpinnings of the modeled variable, which we termed cost evidence (see Figure 2 for an overview). The task involved participants squeezing a handgrip with a given force, knowing that their payoff will be proportional to their effort duration. Cost evidence can be manipulated by varying either an imposed duration or an imposed force (task difficulty). In a first study, we used three tasks that impose variable durations in order to verify that the behavior is adapted on the fly due to internal constraints (bounds). In a second study, we demonstrate that explicit ratings of subjective exhaustion do not follow the cost-evidence variable that accounts for the decision to stop effort exertion. In a third study, we used three other tasks that vary the difficulty in order to dissociate the effects of expected and actual costs.

## Results

### Behavioral adaptation to cost evidence (study 1)

In our previous paper [10], we suggested that the alternation of effort and rest periods observed in the Effort Allocation Task was well explained by a waning and waxing accumulation signal. However, this cost-evidence signal that we localized in the brain could be epiphenomenal, in the sense that it would not reflect any causal mechanism triggering the decisions to stop and restart effort. In this first study, we wished to verify that the level of cost evidence imposes actual constraints on subsequent behavior, as predicted by the accumulation-to-bound principle. We therefore tested the predictions of the accumulation model on the behavior that followed an effort whose duration was imposed. The difficulty was not manipulated in this study, for two reasons: firstly, the effect of difficulty was already shown in the previous paper [10] and will be further investigated in the following studies, and secondly, manipulation of difficulty only applies to effort periods, whereas manipulation of duration can be equally applied to both effort and rest periods. Predictions of the accumulation model are that 1) prolonging effort should decrease the next effort period (if compensatory resting is not allowed), 2) prolonging rest should increase the next effort period (up to a maximum corresponding to full recovery), and 3) prolonging effort should increase the next rest period (if compensatory resting is allowed). These three predictions were tested in different groups of participants (n = 36 in total), using three variants of the Effort Allocation Task. These three Adaptation Tasks had the same structure, with first an imposed effort (between start and stop signals), second a rest period (either fixed or free) and third a free effort exertion. Difficulty of both efforts was fixed at 60% of the maximal force, and payoff was proportional to the duration of the last effort, which was the main dependent measure. Data were regressed at the individual level against a linear model that included the factor of interest (the imposed duration) and several potential confounds (see methods). The statistical significance of regressors was estimated at the group level using two-sided one-sample t-tests. Results are given as standardized effect size (beta) ± inter-subject standard error of the mean.

### Effort is adapted to accumulated cost evidence (Task 1)

**Figure 3:**
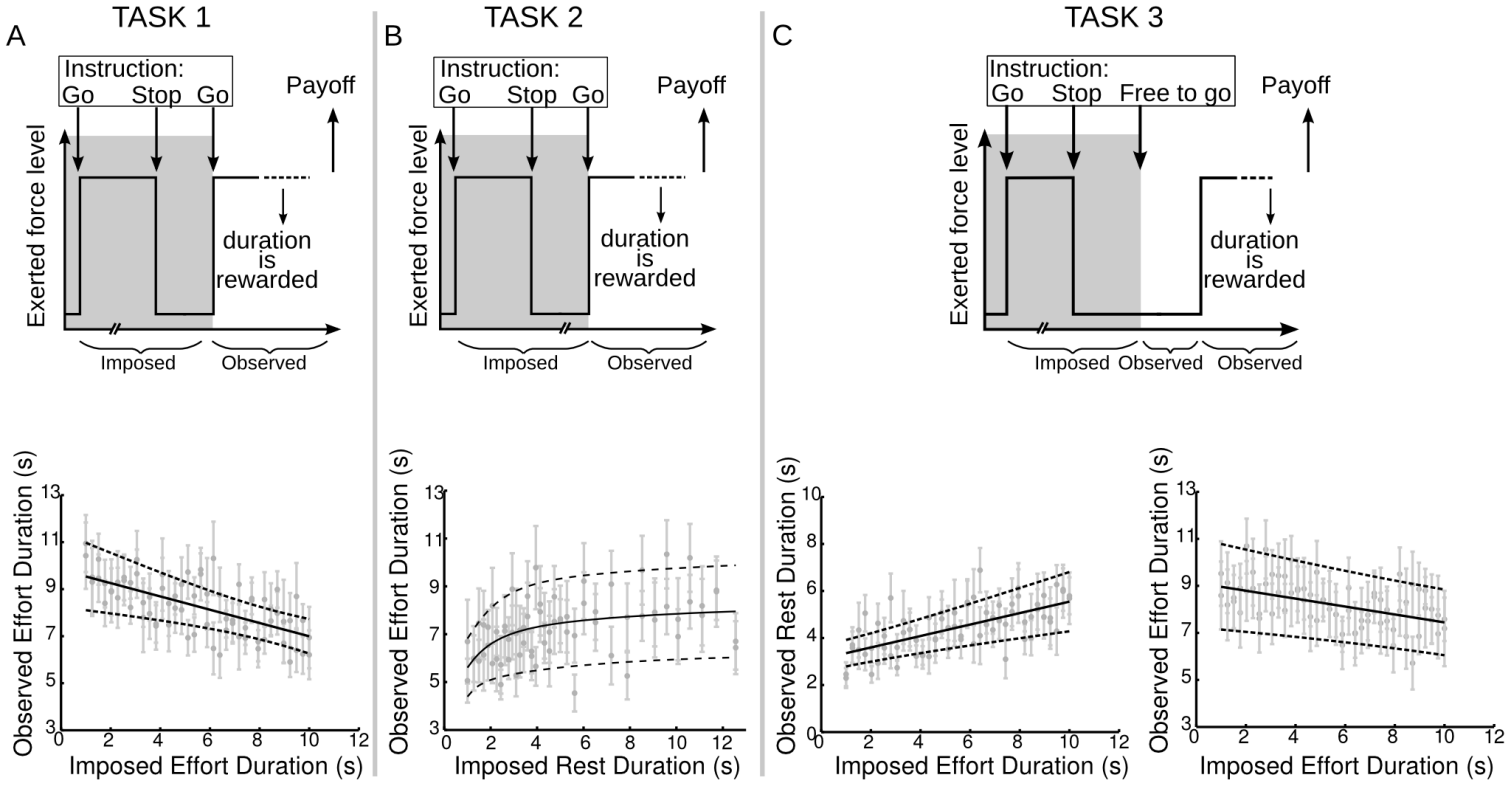
Behavioral adaptation to cost evidence. The three columns (A, B and C) present three different studies, with results underneath the tasks. Note that there are two sub-columns for the last study (on the right) because there are two dependent variables (rest and effort duration). Top: Behavioral tasks. Each plot sketches the variations of exerted force level within a trial. Gray shading indicates the periods when action was imposed on participants; in the other periods the behavior was let free. The broken line points to periods when durations were systematically varied. Bottom: Relation between imposed and observed group-level average durations (± SEM). Two data points are plotted for each imposed duration, corresponding to left and right hands. The black line is the group average of the model fit estimated at the subject level; dash lines indicate the 95% confidence interval of the average.

In this task, cost evidence was increased by prolonging the first effort period (from 1 to 10s), then the second effort duration was observed after a fixed 2-s rest (Figure 3A). To ensure that the rest duration was well controlled, we checked that initiation delay of the second effort after the go signal was not significantly impacted by the duration of the first effort (6.0 10^−2^±3. 2 10^−2^, df = 11, p = 0.09), by cumulated duration of efforts produced in the current session (1.1 10^−2^±3.4 10^−2^, df = 11, p = 0.76), and by the session number (−2.8 10^−2^±2.3 10^−2^, df = 11, p = 0.25). Critically, the second effort was significantly shortened by prolonging the first effort (−8.29 10^−1^±2.3 10^−1^, df = 11, p = 0.0037).

Next we examined the shape of the transfer function from imposed to observed effort duration. The model predicts that this link should be negative, except if resting is long enough to fully dissipate the accumulated cost. We therefore compared a model with pure negative correlation (no saturation, #1) to models with an upper plateau (over shortest efforts), followed by a decrease. We tried two possibilities for this saturation effect: first a constant followed by a linear decrease (#2) and second a negative exponential (#3). The latter was implemented because it provides a better fit of plateau effects when data are noisy (see methods). Bayesian model selection revealed that the pure linear model was far better than the two saturation models in the family comparison (model 1 versus models 2 & 3), with an expected frequency ef = 0.81 (which is much higher than chance level - 1/2) and an exceedance probability xp = 0.96 (confidence that the model is more frequently followed than the others). Thus, the result supports linear accumulation of cost evidence, which limits subsequent effort production due to the existence of an upper bound. However, we found no evidence for the existence of a lower bound in cost dissipation, probably because our rest period was not long enough. This limitation was overcome in the next task, where rest period was systematically varied.

### Effort is adapted to dissipated cost evidence (Task 2)

This task (Figure 3B) was very similar to Task 1, except that effort duration was now fixed (to 7 s) and rest duration was systematically varied (from 1 to 12 s). We checked again that subjects were not delaying effort initiation to compensate for variations in the imposed rest duration (−3.7 10^−2^±2.1 10^−2^, df = 11, p = 0.10). In addition we found that the initiation delay was slightly affected by the cumulated duration of past efforts (1.2 10^−2^±5.0 10 ^−3^, df = 11, p = 0.03), but not by the session number (−1.1 10^−2^±1.4 10^−2^, df = 11, p = 0.46). Critically, observed effort was significantly prolonged by longer rest (6.9 10^−1^±1.9 10^−1^, df = 11, p = 0.0035).

Next we tested the existence of a saturation, meaning that beyond a certain rest duration, cost evidence is entirely dissipated and subsequent effort cannot be further prolonged. As was done for the previous task, we compared three models for the link between rest and effort duration: 1) a linear effect (no saturation), 2) a linear effect bounded by an upper plateau (over longest rests), 3) an exponential asymptotic plateau. Bayesian model selection showed that the saturation family was now more plausible (models 2 and 3 versus model 1, chance level is 1/2, ef = 0.79, xp = 0.94). Direct comparison between models 2 and 3 revealed that the asymptotic saturation was more likely than the linear plateau (xp = 0.98). Thus, the results confirmed that prolonging rest after a first effort augments the capacity to produce a second effort, as if cost evidence was dissipated. Moreover, the saturation effect suggests the existence of a threshold after which prolonging rest is useless, which would correspond to a lower bound for cost-evidence dissipation.

### Rest is adapted to accumulated cost evidence (Task 3)

This task (Figure 3C) was quite similar to Task 2, except that participants were not asked to resume their effort immediately at the go signal, but only when they felt ready to do so. There were therefore two dependent variables of interest: rest duration and subsequent effort duration. Critically, rest duration was significantly increased by prolonging the imposed effort duration (6.5 10^−1^±1.3 10^−1^, df = 11, p = 0.0005).

We expected that participants would rest long enough to fully dissipate the first effort cost, which hence would have no impact on the second effort duration. This was not the case: prolonging the first effort significantly shortened the second effort (−4.7 10^−1^±1.4 10^−1^, df = 11, p = 0.006). Thus, subjects did not wait long enough to compensate for the imposed effort cost. This partial recovery might be related to the fact that the total time allowed for rest and effort was limited to 20s, so that participants may have shortened rest to make sure there would be enough time for effort (even if in reality, 20s was largely enough to fully dissipate and accumulate cost again).

### Introspection of cost evidence (study 2)

**Figure 4:**
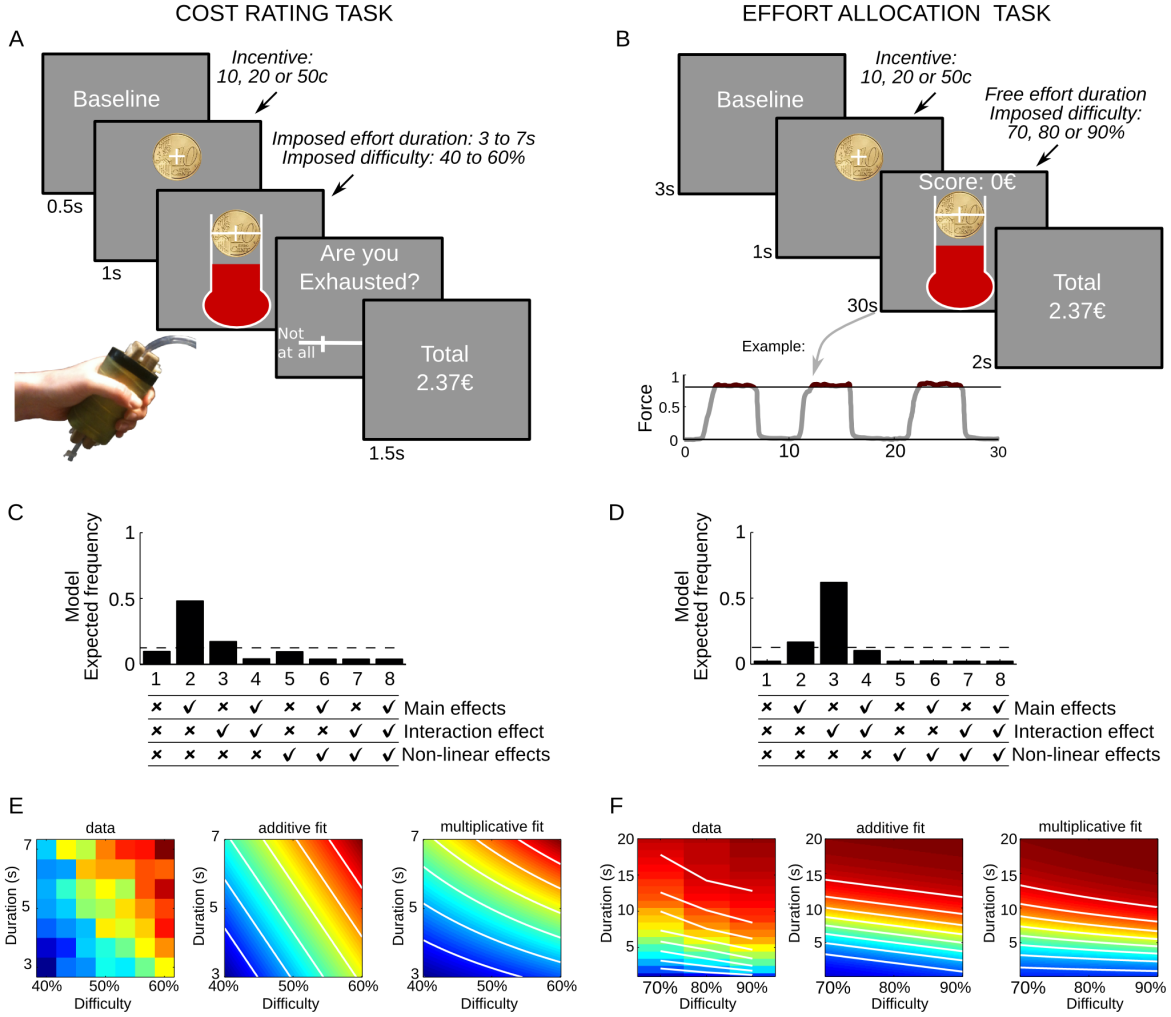
Introspection of cost evidence. A–B: Behavioral tasks. The illustrated screenshots were successively presented every trial. A: the Cost Rating Task was developed to assess introspection of resource exhaustion. On each trial, participants were asked to squeeze the hand grip up to the target level (horizontal bar), which corresponded to varying difficulty level (40% to 60% of maximal force), as long as the thermometer was displayed, which could last for varying durations (4 to 7 seconds). After each effort, participants rated their degree of exhaustion using a visual horizontal analog scale. The last screen of each trial indicated the payoff cumulated over preceding trials. B: the Effort Allocation Task was exploited in a previous paper [10]. When the thermometer image was displayed, participants could squeeze a handgrip to win as much money as possible. Subjects were provided with online feedback on force level and cumulative payoff. The payoff was only increased when force level was above the target bar, at a constant rate proportional to the monetary incentive. The incentive (10, 20 or 50 cents) and the difficulty (i.e. the force required to reach the target bar: 70, 80 or 90% of maximal force) were crossed over trials. The last screen indicated the money won over all preceding trials. C–D: Computational modeling: Bayesian model comparison. For each participant, we estimated eight models generating cost evidence from difficulty and duration. Cost evidence was then used to fit the subjective ratings of exhaustion (C) or the decisions to stop effort exertion (D). Models were linear combinations of different possible regressors (main effects, interaction and non-linear effects), as indicated in the bottom chart (tick: included, cross: not included). E–F: Fit of additive and multiplicative models. Data are subjective ratings of exhaustion (E) and probability of stopping effort exertion (F), shown in the duration by difficulty space explored in the Cost Rating and Effort Allocation tasks, respectively. The color code indicates average observed data (left diagram) or predicted data from the additive and multiplicative models that provided the best fit with median parameter values (middle and right diagrams). Note the main difference between additive and multiplicative models is the curvature of iso-value lines (in white), which reflects the interaction between duration and difficulty.

So far, our results suggest that effort duration is not entirely planned in advance but adapted on the fly so as to keep cost evidence within pre-defined bounds. The next study was designed to assess whether our participants could explicitly report the cost evidence that was monitored by their brain in order to regulate their behavior. The first study only manipulated the duration of effort or rest periods. Yet our model posits that cost evidence accumulation during effort depends on task difficulty. Therefore, cost-evidence level should reflect the interaction of task difficulty and effort duration. The logic of this second study was first to examine whether introspective reports would reflect the interaction of difficulty and duration, and then to verify that behavioral choices were indeed driven by this interaction,

For introspective reports we asked a new group of 18 participants to perform a Cost Rating Task, in which they had to rate their degree of exhaustion after effort exertion. Note that we could have directly inserted cost ratings within the Effort Allocation Task, but subjects in this case might have artificially aligned their behavior to their explicit reports (or vice-versa). Another issue with this possibility was that effort duration would not have been sufficiently varied, at least not orthogonally to effort difficulty, since subjects would have stopped their effort when cost evidence (difficulty times duration) reached a pre-defined bound. We chose to frame the question in terms of exhaustion because debriefing of previous studies revealed that exhaustion is the intuitive term that subjects spontaneously use to describe the sensation that makes them cease their effort. The precise question was ‘Have you exhausted your resources?’ and the response scale was ranging from ‘not at all’ to ‘completely’. In this Cost Rating Task, both effort duration (from 3 to 7 s) and task difficulty (from 40 to 60% of maximal force) were imposed and varied experimentally (Figure 4A). To keep similarity with the Effort Allocation Task, we also manipulated the incentive level. Yet we acknowledge that the comparison between tasks has limitations, first because they implement different range of forces and durations, second because they are performed by different subjects, who might have different sensitivity to effort cost.

On each trial, the payoff was calculated as the incentive multiplied by the fraction of the imposed duration that subjects spent squeezing at the required target force level or higher. As participants were asked to be as accurate as possible, this fraction was almost 100% (mean over subjects: 98.7%, extreme subjects: 94.6% and 99.9%). The difference between required and produced force levels did not vary significantly across conditions (multiple regression analysis and two-sided t-test with df = 17; incentive: 4.1 10^−3^±3.7 10^−3^, p = 0.28; duration: −3.4 10^−6^±4.6 10^−3^, p = 0.99; difficulty: −1.8 10^−3^±3.3 10^−3^, p = 0.59; interactions between these factors: all p>0.21), suggesting that effort production was well controlled by the experimental design. Cost ratings were not significantly impacted by incentives (1.4±0.86, df = 17, p = 0.1), and marginally by the initial position of the cursor on the scale (1.8±0.9, df = 17, p = 0.056). Critically, cost ratings increased with both duration (1.9±0.79, df = 17,p = 0.028) and difficulty (3.2±0.49, df = 17, p = 5 10^−6^), without significant interaction between these factors (p = 0.96).

We then fitted cost ratings with a linear combination of regressors meant to capture the impact of duration and difficulty. We considered three possibilities: main effects of duration and difficulty, non-linear effects (power functions) of duration and difficulty, and interaction between duration and difficulty. Including or not each possibility in the linear combination made a total of eight models, which we compared using Bayesian model selection (Figure 4C). This analysis confirmed the absence of significant interaction between duration and difficulty, since the best model was simply additive (chance level is 1/8, ef = 0.48; xp = 0.93). In principle, this additive effect could arise from half the subjects reporting duration and the other half reporting difficulty. This would imply that the effect sizes of these factors are anti-correlated across subjects. We found the opposite result (Pearson rho: 0.82, df = 16, p = 3 10^−5^), suggesting that subjects who were good at perceiving duration were also good at perceiving difficulty. Yet they reported the addition of the two dimensions, and not their product, as should be the case if they were simply introspecting cost evidence.

We next re-analyzed the behavioral choices observed in our Effort Allocation Task (Figure 4B) that involved subjects (n = 38) squeezing a handgrip in order to accumulate as much money as possible [10]. The payoff was calculated as the monetary incentive multiplied by the time spent above a target force level (which indexed task difficulty). Both the incentive (10, 20 or 50 cents) and difficulty levels (70, 80 or 90% of maximal force) were varied across trials such that we could assess their effects on effort allocation. Incentive levels were sufficient for subjects to initiate the effort and to reach the target, but difficulty levels were too demanding for subjects to sustain their effort throughout trials, which lasted 30 seconds. Instead, they freely alternated effort and rest periods within trials (as can be seen in Figure 1A). We used the normalized cumulative distribution of effort durations to calculate the probability of stopping the effort after a given duration at a given difficulty level. This probability was fitted with a sigmoid function of cost-evidence level, which accounts for higher cost evidence making effort cessation more likely. Cost evidence was then modeled with the same linear combinations as used for fitting cost ratings. Results of Bayesian model selection (Figure 4D) showed that the most plausible model was pure interaction (chance level is 1/8, ef = 0.62, xp = 0.988).

The Cost Rating and Effort Allocation tasks thus elicited distinct forms of cost evidence, with additive versus multiplicative effect of effort difficulty and duration. The critical difference is the shape of iso-value lines of cost evidence in the duration by difficulty space, with straight lines for explicit report and convex lines for effort cessation (compare Figures 4E and 4F). To directly compare the curvature of cost evidence inferred from introspective reports and behavioral choices, we fitted a model with constant elasticity of substitution (CES) between duration and difficulty (see methods). This model has a free parameter that captures the curvature of cost in the duration by difficulty space, which should be equal to one in the absence of interaction, and below one in the case of a convex interaction. We found that the curvature parameter was significantly below one in the Effort Allocation Task (median: 0.52, SEM: 0.06; two-sided sign-test of the median against 1: p = 6.7 10^−8^) but not in the Cost Rating Task (median: 1.01, SEM: 0.12; sign-test of the median against 1: p = 1), with a significant difference between tasks (p = 3 10^−6^, two-sided Wilcoxon rank sum test for equal medians).

When debriefing the Cost Rating Task, participants unambiguously reported having noticed variations in both difficulty and duration. When asked whether one of these two factors had a greater impact on their ratings, 13 subjects favored the duration, 3 favored the difficulty, and 2 could not favor one or the other, describing something like an interaction. However, comparison of standardized effect size revealed a greater impact of difficulty on ratings (paired t-test on duration minus difficulty effect size: −1.3±0.48, df = 17, p = 0.016). Among the 16 subjects who favored a main effect, 12 got it wrong (the other factor had a higher impact on their ratings), which is more than expected by chance (binomial test, p = 0.028).

To summarize, the costs reported in subjective ratings do not have the same shape as the costs inferred from behavioral choices. What subjects report is an addition of duration and difficulty, whereas what drives their behavior is an interaction between the two. Furthermore, at a meta-cognitive level, subjects have poor insight into the factors that modulate their sensation of exhaustion.

### Dissociation of implicit from explicit cost processing (study 3)

The two studies presented so far are compatible with a completely implicit and automatic model, in which decisions to cease and resume effort production are controlled by an internal variable fluctuating between bounds that might be determined by physiological constraints. In this last study, we explored whether explicit information about cost could impact the mechanics driving decisions to start and stop effort exertion. In our previous paper [10], we had observed that task difficulty shortened effort duration, which could reflect cost evidence (difficulty times duration) reaching the upper bound, but did not affect rest duration. We hypothesized that the last observation could arise from task difficulty not being made explicit to participants. Indeed, monetary incentives, contrary to difficulty levels, were explicitly presented with coin images at trial start and affected both effort and rest durations (with longer effort and shorter rest for higher incentive).

We therefore tested whether providing explicit information about difficulty level would change the way participants process cost evidence. We constructed three variants of the Effort Allocation Task, which were administered to three different groups of participants (n = 67 in total). In all tasks, incentives (coin images) were explicitly displayed before and during trials, which had a fixed duration (30s) that was specified to participants prior to the experiment. The Implicit Task (Figure 4B) is the task used in our previous paper [10], with no visual cue for difficulty level. In the Explicit Task, the only change is that difficulty level (percentage of maximal force: 70, 80 or 90%) was announced before the beginning of trials, on the same screen as incentive level. In the Dissociation Task, we kept the explicit cues, but they were no longer predictive of the actual task difficulty. To maintain sufficient statistical power, only two difficulty levels were used (75 and 85%), in a full factorial design (two cued difficulties crossed by two actual difficulties). This design was meant to disentangle the effects of implicit versus explicit cost processing. Monetary incentives were also manipulated in all tasks and crossed with the three (Implicit and Explicit Tasks) or four (Dissociation task) cells corresponding to variations in difficulty. We only used two incentive levels (10 versus 20c) in the Dissociation task to avoid combinatorial inflation. In every task, the effect of experimental factors (incentive, actual and cued difficulty) on the duration of effort and rest epochs were estimated in separate multiple linear regressions followed by two-sided one-sample t-tests.

Note that because they must add up to 30s, the cumulative durations of effort and rest are anti-correlated. However, this dependency was broken first because the last rest epochs were discarded from the analysis, since they are interrupted by trial ending, and second because we considered the single epoch durations, which are not predictable from the cumulative durations, since they depend on the number of alternations between effort and rest. The remaining correlation was rather low (Pearson rho: −0.15±0.026 in the main Implicit Effort Allocation Task) and probably due to opposite effects of experimental factors (see below).

### Comparison of Implicit and Explicit Tasks

**Figure 5:**
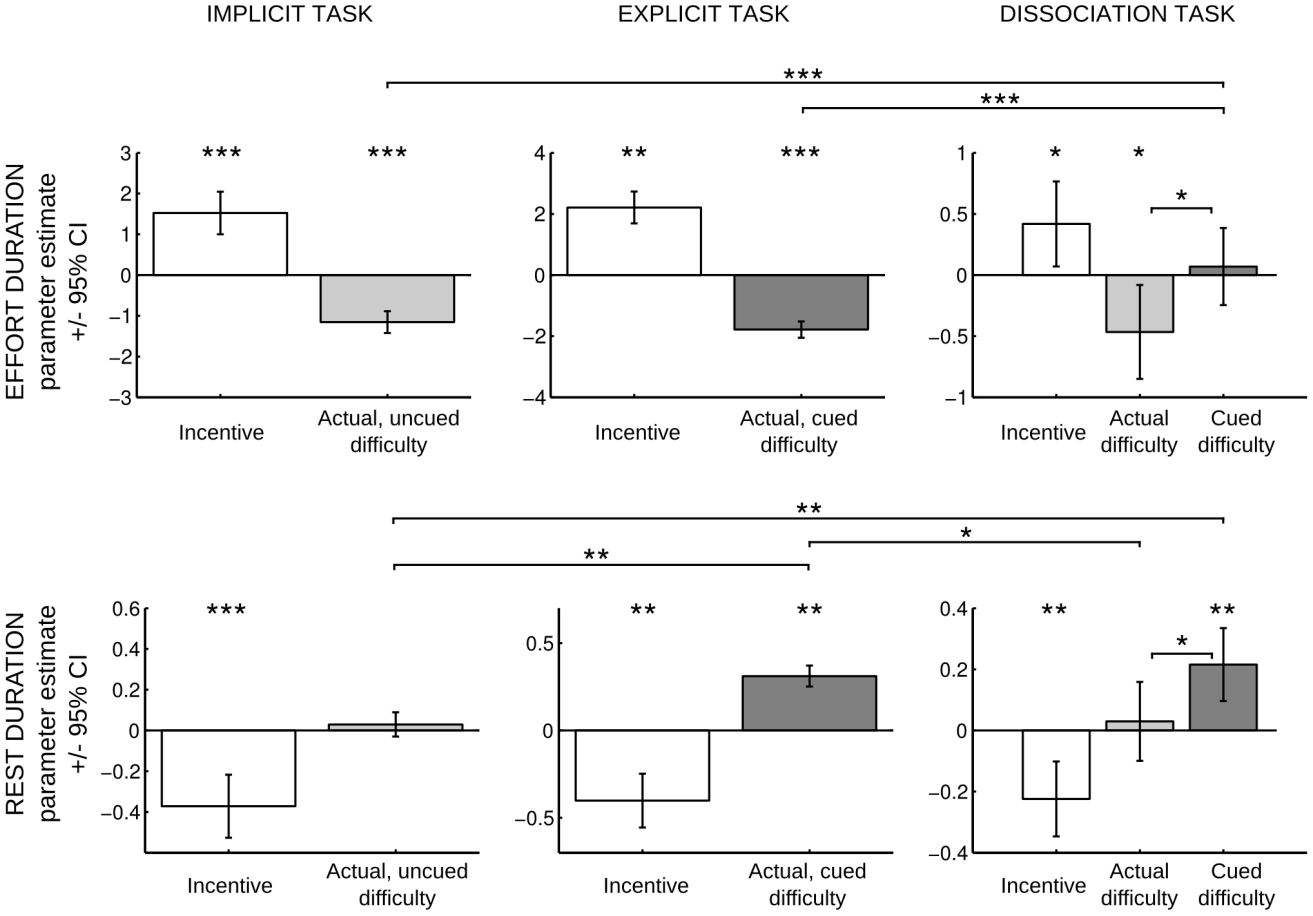
Dissociation of implicit from explicit cost processing. Three sets of participants performed three slightly different versions of the Effort Allocation Task. The Implicit Task is sketched in Figure 4B. The only variation introduced in the Explicit Task is that effort difficulty was written on screen (70%, 80%, 90%) along with incentive level, announced as a coin image. The Dissociation Task was visually identical to the Explicit Task, the difference being that the difficulty level announced on screen was not predictive of the actual difficulty level: cued and actual difficulties were crossed into a factorial design. Thus, the factorial combination generated 9 cells for the Implicit and Explicit Tasks (3 incentives×3 difficulties) and 8 cells for the Dissociation Task (2 incentives×2 actual×2 cued difficulties). The effects of the different factors, estimated with linear regression analysis, are illustrated column-wise for each task. Regression coefficients were statistically tested and compared with two-sided t-test, p-values: *<0.05, **<0.005, ***<0.0005.

As previously shown [10], in the Implicit Task (Figure 5, left), effort duration was both longer for higher incentive (1.5±0.26, df = 37, p = 8.1 10^−7^) and shorter for higher difficulty (−1.1±0.13, df = 37, p = 1.6 10^−10^). In contrast, rest duration was shorter for higher incentive (−0.37±0.08, df = 37, p = 2.0 10^−5^) but was not modulated by the difficulty (0.03, ±0.03, df = 37, p = 0.32). Interactions were included in the regression model, but the incentive x difficulty interaction was not significant, neither for effort or for rest duration (all p>0.084).

All significant results were replicated in the Explicit Task (Figure 5, middle): effort duration was both longer for higher incentive (2.2±0.53, df = 13, p = 1.1 10^−3^) and shorter for higher difficulty (−1.8±0.24, df = 13, p = 6.0 10^−6^), and rest duration was shorter for higher incentive (−0.4±0.09, df = 13, p = 9.7 10^−4^). The novel result is that rest duration was now increased by higher difficulty (0.31±0.08, df = 13, p = 1.6 10^−3^), which was correctly cued at trial start. The difference in standardized effect sizes between Implicit and Explicit Tasks was also significant (p = 1.2 10^−4^, unpaired t-test, df1: 37, df2: 13). All interactions remained non-significant, neither for effort or rest duration (all p>0.1). Thus, the difficulty in the Explicit Task, which was both expected and experienced during effort exertion, affected both effort and rest durations.

The results obtained with the Implicit and Explicit Tasks are compatible with the actual difficulty affecting effort duration, and the expected difficulty affecting rest duration. In the Implicit Task, there was no explicit cue, so subjects did not expect any particular difficulty level, and consequently only effort duration (not rest duration) was affected by task difficulty. In the Explicit Task, both effort and rest durations were modulated because the actual difficulty was fully expected. However, as the explicit cues were perfectly valid, we could not formally demonstrate with this task that rest duration is not concerned with actual difficulty, or that effort duration is not concerned with expected difficulty. To complete our demonstration, we intended to dissociate the two effects within the same task.

### Analysis of the Dissociation Task

In the Dissociation Task (Figure 5, right), the levels of actual and cued difficulty were manipulated independently. As in the Implicit and Explicit tasks, higher incentive increased effort duration (0.42±0.16, df = 14, p = 0.022) and shortened rest duration (−0.22±0.06, df = 14, p = 1.5 10^−3^). Effort duration was affected by the actual (−0.47±0.18, df = 14, p = 0.021) but not by the cued difficulty (0.07±0.15, df = 14, p = 0.64). The difference in standardized effect size was at significance limit (−0.54±0.25, df = 14, p = 0.050, paired t-test). We also verified that the effect of cued difficulty on effort duration in the Dissociation Task was significantly lower than the (actual) difficulty effects observed in the Implicit (p = 4.3 10^−7^, unpaired t-test, df1: 37, df2: 14) and Explicit (p = 2.3 10^−6^, unpaired t-test, df1: 14, df2: 13) tasks. Conversely, rest duration was affected by the cued (0.22±0.06, df = 14, p = 1.7 10^−3^) but not by the actual (0.03±0.06, df = 14, p = 0.63) difficulty. The difference in standardized effect size was as well significant (−0.19±0.08, df = 14, p = 0.045, paired t-test). We also verified that the effect of cued difficulty on rest duration was higher in the Dissociation Task than the (actual) difficulty effect observed in the Implicit Task (p = 0.002, unpaired t-test, df1: 37, df2: 14), and that the effect of actual difficulty in the Dissociation Task was lower than the (cued) difficulty effect observed in the Explicit Task (p = 0.008, unpaired t-test, df1: 14, df2: 13). Thus, within- and between-task comparisons both support a double dissociation between the actual and cued difficulty effects on effort and rest durations.

As some critical p-values were near 0.05 type I error rate, we conducted a permutation test to ensure the reliability of the parametric t-distribution in our small sample. This permutation-based t-distribution yielded the same exact p-values up to the 3^rd^ decimal. Second and third order interaction terms between incentive, cued and actual difficulty were included in the model, but none of them was significant neither for rest or effort duration (all p>0.18). We also checked that there was no interaction of cued difficulty with time, which could potentially reflect a progressive discount of the cue effect (as subjects would learn that cues are not predictive of actual difficulty). Time was modeled at three nested scales (rest or effort period position within a trial, trial position within a session, and session number). Two-way interactions with cued difficulty were estimated for each time scale: none of them was significant (all p>0.25).

### Bayesian model selection

**Figure 6:**
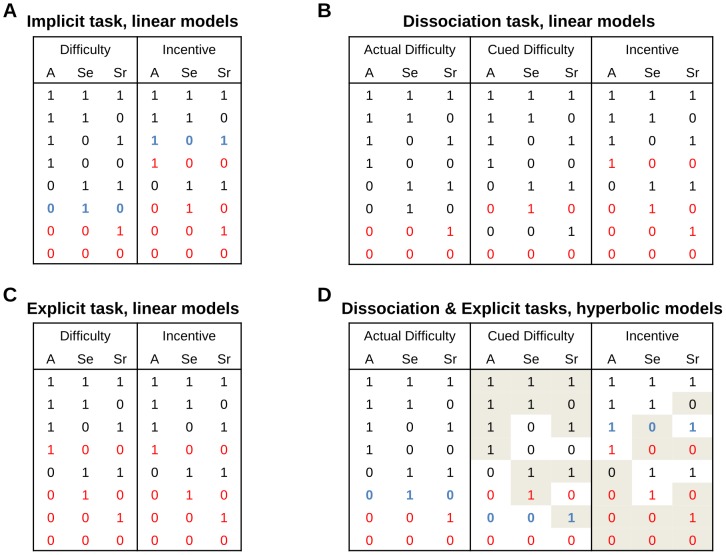
Model space definition. Models are characterized by the modulation of latent parameters (A: amplitude between bounds, Se: accumulation slope during effort, and Sr: dissipation slope during rest) by the experimental factors (incentive, actual difficulty, cued difficulty). Each column is a potential factor and each line is a possible set of modulations by this factor (‘1’ denotes that the modulation is allowed, ‘0’ that the modulation is absent). For each latent parameter, the different modulations are linearly combined (models A, B, C), except for the cued difficulty in hyperbolic models (D), which is integrated as a hyperbolic discounter of monetary incentives. In this case, including cued difficulty (in the denominator) is useless when modulation by incentives (the numerator) is not allowed, which is indicated by a gray background. Lines appearing in red correspond to models that were not included in the comparison, because they cannot produce the behavioral results (significant effect on effort or rest duration). The winning models for the different tasks appear in blue (note that it is indeed the same model, as there is no cued difficulty, and hence no hyperbolic discounting, in the Implicit Task).

We compared different versions of our accumulation model to identify how the latent parameters (A: amplitude between bounds, S_E_: accumulation slope during effort, and S_R_: dissipation slope during rest) were affected by the experimental factors (I: Incentive, Da: actual difficulty, Dc: cued difficulty). We started with the formalization that we proposed in our previous publication [10] to account for the behavior observed in the Implicit Task. All models were built as a set of three equations that defines each latent parameter as a linear combination of the different factors (see methods). Only models that can produce the behavioral results (significant effect on effort or rest duration) were included in the space covered by Bayesian Model Selection. In the Implicit Task, this left 24 possible models (see Figure 6A) with one that was much more plausible than the others (chance level is 1/24, ef = 0.30, xp = 0.90).

For the novel tasks (Explicit and Dissociation), we explored two possibilities for integrating the additional factor (cued difficulty). The first possibility was to integrate it as an additive term, just as was done with actual difficulty (see Figure 6B and 6C). Note that these purely linear models do not enable dissociating the effects of actual and expected difficulty in the Explicit Task. The second possibility was to integrate cued difficulty as a hyperbolic discounter of incentives, which is quite standard in the literature for capturing temporal discounting [11]–[13]. Thus, for the novel tasks that manipulate expected difficulty, we included the hyperbolic equivalent of our linear models (see Figure 6D). With this hyperbolic version, we can dissociate the effect of actual and expected difficulty (the former is linear, the latter hyperbolic) even in the Explicit Task where the two factors are confounded.

Family comparison revealed that there was far more evidence in favor of a hyperbolic rather than linear discount of incentives by cued difficulty, in both the Explicit and Dissociation tasks (chance level is 1/2, ef>0.91, xp>0.999). Among the 78 possible hyperbolic models, a best model was identified with xp = 0.90 (chance level is 1/78, ef = 0.13) in the Dissociation Task and with xp = 0.82 (chance level is 1/78, ef = 0.14,) in the Explicit Task. Crucially, the best hyperbolic model identified in the Explicit and Dissociation tasks was the same model, which also corresponded to the best model identified in the Implicit Task (where modulation by cued difficulty is necessarily absent). This best model is written as follows (Te and Tr being effort and rest duration, α, β, γ the coefficients and I, Da and Dc the incentive, actual difficulty and cued difficulty levels):
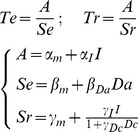
A graphical interpretation of the model with a summary of the observed effects is provided in Figure 1. In short, incentives impacted both the amplitude between bounds and the dissipation rate, resulting in longer effort and shorter rest for higher incentives. The effect of task difficulty was computationally dissociable: higher actual difficulty accelerated the accumulation, resulting in shorter effort, whereas higher expected difficulty slowed the dissipation, resulting in longer rest.

## Discussion

In our previous paper [10], we addressed the issue of how the brain allocates effort production over time, in a situation where the payoff depends on the total effort duration. We found a neural signal that was ramping up and down during effort and rest periods and that could, in principle, trigger the decisions to stop and restart effort production. Here we provide evidence that the core accumulation-to-bound mechanism is reactive and implicit. Indeed, participants adapted their behavior on the fly when we implicitly manipulated both the duration (Study 1) and the difficulty (Study 3) of effort exertion. However, when asked to rate their degree of exhaustion (Study 2), subjects did not report the cost evidence signal that was shown to drive their behavior. In addition, we suggest that some latent parameters of the accumulation-to-bound process are susceptible to anticipatory adjustment based on explicit information. Indeed, we found that expected benefit and difficulty could modulate the distance between bounds or the dissipation rate during rest. The dissociation of implicit and explicit cost processing could reconcile the perspectives offered by sport physiology on the one hand, and economic theory of choice on the other hand.

The implicit part of the model - monitoring cost evidence and triggering decisions when bounds are attained, accords well with the literature on exercise performance [4], [8]. Although it was developed to explain how athletes pace their running on treadmills, we can borrow the notion that behavioral changes are reactions to physiological variables reaching homeostatic borders. Results of Study 1 show that bounds between which the cost-evidence signal fluctuates are true limits that determine the decisions to stop and restart effort exertion. On the contrary, the explicit part of the model – adjusting the behavior depending on expected benefit and difficulty – is consistent with the literature on value-based decision-making [14], [15]. It is quite remarkable in Study 3 that the computational effect of actual (implicit) difficulty during effort was simply linear, as in a passive accumulation, whereas the effect of expected (explicit) difficulty was hyperbolic, as in economic models of temporal discounting [13], [16]. It should be acknowledged that mixtures of anticipatory calculations and on-line adaptations are frequently used in motor control theory [17], [18], for instance to explain how movement trajectory can be adjusted to internal noise and to unexpected target displacement. However, these models have not integrated the conflict between costs and benefits until very recently [19], [20]. Finally, we note that the perspectives offered by the literatures on exercise performance and value-based choice only explain the duration of effort; without further specification they say nothing about the duration of rest. Our model accounts for the timing of both effort and rest, within the same accumulation framework.

Examining whether the accumulation mechanism is optimal or not would go beyond the scope of this study. It can nonetheless be seen has a heuristic mechanism that certainly has advantages. Physiologically, it ensures that effort production does not put the body at threat, avoiding for instance damage to the muscles. In this view the signal would indicate the likelihood of physiological damage, and the upper bound would implement a threshold on that risk. Economically, it ensures that costs do not overcome benefits. In this view, the signal would indicate the cost, and the upper bound the benefit of the potential effort at the next time point. Mixing predictive and reactive processes also presents advantages. Online monitoring of effort consequences allows refining cost estimation, which is usually uncertain beforehand, as in our implicit version of the task. Anticipatory estimation allows deciding whether or not to engage the action, and scaling energy expenditure to expected costs and benefits. In our case, this means spending more time at work and less time at rest when the net value of effort is higher.

The two behaviors, effort and rest, are not equivalent though. While monitoring cost evidence during effort might be a passive process (mechanically integrating difficulty over time), dissipating cost evidence during rest seems more active. Indeed, the dissipation rate was susceptible to modulation by explicit information (monetary incentive and cued difficulty). Moreover, the observation that subjects do not report cost evidence was only made in Study 2, relative to the effort period. It remains possible that during rest, subjects are fully aware of the cost-evidence level, and hence of how much effort they would be able to produce next. We could have tried to test whether their introspective reports integrate duration with cued difficulty after a given rest, but asking the question in this case would have been awkward.

Using dissipation as well as accumulation in order to explain behavioral choices is a major difference between our model and the standard evidence accumulation models. Classically, accumulation of evidence is meant to improve the estimation of a stationary noisy input, whether external, as in perceptual decision-making [21]–[23], or internal, as in value-based decision making [24]–[26]. The fact that the cost evidence variable dissipates at rest rules out the possibility that this signal simply reflects an integration of the force produced throughout the trial (which can only increase). It is likely that the signal reflects an input that is already dynamical (and not stationary). This might be true not only at the theoretical level, if we interpret it as signaling the potential effort cost or the proximity of exhaustion, but also at the biological level. For instance, our cost evidence signal could relay the accumulation and dissipation of a by-product of effort exertion, which could integrate several variables such as lactate concentration, stretch of muscle fibers or heart beat rate. Alternatively, the cost-evidence signal could reflect increase in the efferent drive needed to overcome fatigue and maintain motor output [27]. Using combined fMRI and MEG recordings, we localized the cost-evidence signal in proprioceptive areas (posterior insula). This localization would incline us to situate the input in the afferent proprioception coming from the muscles [28]. However, the fact that subjects had a poor introspection into that signal argues against the idea that it represents the neural counterpart of a common and intuitive sensation such as fatigue.

Yet the fact that cost evidence dissipation could be modulated depending on expected benefit and difficulty suggests that other neural processes occur during rest than passive transmission of effort-induced physiological perturbation. First, the dissipation of cost evidence could be linked to the preparation of the next effort. Such preparation is reflected by motor signals such as the readiness potential [29], [30] or the de-synchronization of beta oscillations [31], [32]. We showed in a previous publication [33] that the last process is modulated by incentive level; it could therefore mediate the effect of motivation on cost dissipation in the posterior insula. Second, the dissipation of cost evidence could be accentuated by analgesic mechanisms. The posterior insula region that signals cost evidence is also involved in pain perception [34], [35] and placebo effect [36]. The placebo effect suggests that the brain has an endogenous means to control pain, possibly through the opioid system [37]–[39]. Another possibility would be serotonin, which contributes to the analgesia induced by common pain killers such as acetaminophen [40] and to the sensation of fatigue during effort [1], [5]. Thus, through opioids or serotonin, the brain might be able to regulate cost-related signals depending on motivation level.

Before concluding, we must acknowledge some limitations and inconsistencies. First, the situation explored in our paradigm is highly restricted. Notably, subjects are only allowed to adjust the duration of their effort and not the intensity, which we can usually adjust in ecological situations. We have conducted a series of studies where the payoff was based on effort intensity [41]–[43], but we still have to explore a situation where the two dimensions can vary. Second, the model does not account for a number of observations, for instance the fact that fatigue accumulates at longer time scales. Indeed, we observed in some tasks an effect of trial and/or session number on effort duration, which could mean that cost was not fully dissipated after each rest period, or that slower effort-induced perturbations were accumulated elsewhere and imposed constraints on performance. Third, it cannot be formally concluded that subjects have no explicit access to the cost-evidence signal driving effort allocation, as one can always object that the inability to report a variable is due to the question not being appropriately formulated. This objection should nevertheless be tempered since our negative result is not an absence of effect: the question about exhaustion did elicit subjective ratings that were sensitive to the cost parameters (effort duration and difficulty) but not in the way that is relevant to cost monitoring (multiplicative and not additive). It remains interesting that, although participants spontaneously explain why they stopped their effort in terms of exhaustion, they failed to report the variable (difficulty times duration) that exhausted their resources.

Despite these limitations, the results of the present studies taken together provide strong evidence that costs are implicitly monitored in order to adapt effort duration on the fly, which can be dissociated from anticipatory adjustments depending on explicit costs and benefits. Moreover, this dissociation was computationally tractable and might be of clinical relevance. It suggests the existence of two different kinds of apathy: effort could be limited because the expected cost is over-estimated, or because the actual effort-induced cost is inflated. The first category (perhaps in depression disorders) would rest a lot but would encounter little difficulty in maintaining their effort once it is engaged, whereas the other (perhaps in chronic fatigue) would easily initiate efforts but then would rapidly renounce.

## Methods

### Ethics statement

The study was approved by the Pitié-Salpétrière Hospital ethics committee (protocol number: 106-07). All subjects were recruited via email within an academic database and gave inform consent prior to participating in the study.

### Participants

The study was approved by the Pitié-Salpétrière Hospital ethics committee. All subjects were recruited via email within an academic database and gave inform consent prior to participating in the study. There was no restriction of handedness, excepted for the original (Implicit) Effort Allocation Task, in which participants were all right handed for neuroimaging purposes. Other inclusion criteria were: age between 20 and 39 years, absence of self-reported psychiatric or neurological history and of current psycho-active substance consumption.

In all studies, participants were told that they would win the money accumulated during the task. In the previous study (Implicit task), the payoff was eventually rounded up to a fixed amount (100€) credited by bank transfer. In all new studies participants were paid in cash at the end of the experiment. The payoff was partitioned into a fixed amount and variable amount depending on the money won during the task. For the Cost Rating Task, the amount earned during the task was eventually down-scaled (divided by 2.48) to fit in a budget of 30€ per subject while maintaining the correspondence between payoff and incentive during the task. Participants were informed about this correction prior to the experiment.

The Implicit task was performed in a MRI scanner for half the subjects and under a MEG helmet for the other half. One subject in the MRI group was excluded from the analysis because of calibration issues. For the Adaptation Task 3, one participant was excluded because of calibration issues and another for cheating (repeated, direct manipulation of the air tube). For the Dissociation Task, one participant was excluded due to an instruction issue: she could not understand the meaning of the percentage displayed on the screen, which indicated the difficulty level in proportion of the maximal force. Two other participants were excluded due to calibration issues. The task-specific information is summarized in Table 1.

**Table 1:** Task-specific information on participants.

Task	Exp.	Period	N after exclusion	N male	N excluded	Mean age ± s.e.m.	Fix (€)	Var (€)	Var (€) range
Adaptation Task 1	FM	03/2012	12	2	0	22.7±0.8	10	10.1	7–13
Adaptation Task 2	FM	03/2012	12	0	0	21.9±0.4	10	9.6	4–15
Adaptation Task 3	FM	03/2012	12	4	2	21.7±0.7	10	10.3	6–15
Cost Rating Task	FM	02/2013	18	7	0	22.2±0.5	0	29.8	29–30
Implicit Task	FM	04–05/2010	38	16	1	24.2±0.65	50	31.6	15–48
Explicit Task	FM	03/2011	14	10	0	23.7±0.4	15	13	8–19
Dissociation Task	LS	10/2011	15	5	3	25.4±0.8	10	6	3–10

‘Exp.’ refers to the author who collected the data. The payoff was fractioned into a fixed amount (‘Fix’) for participation and a variable amount (‘Var’) depending on performance, for which we report the mean value and the range (minimum and maximum) across participants.

### Experimental set up

We used homemade power grips composed of two plastic or wood cylinders compressing an air tube when squeezed. The tube was connected to a transducer converting air pressure into voltage. Thus, grip compression resulted in the generation of a differential voltage signal, linearly proportional to the force exerted. The signal was amplified and digitized by a signal conditioner (CED 1401, Cambridge electronic design, UK) for Implicit, Explicit and Dissociation tasks, and by a homemade device for the Adaptation Tasks and Cost Rating task. The digitized signal was read by a Matlab program (The MathWorks Inc., USA).

### Pre-processing of force data

In the Adaptation Tasks (1 to 3), the effort onsets were identified on-line and used to update the screen displayed to the participants. The effort onset was determined as the first sample exceeding 20% of the participant maximal force.

In the Effort Allocation Tasks (‘implicit’, ‘explicit’ and ‘dissociation’), effort onsets and offsets were identified off-line with an algorithm using the same two criteria for all conditions: 1) force temporal derivative higher than one standard deviation and 2) force level lower (for effort onset) or higher (for effort offset) than half the maximal force. The first rest period started with coin presentation and the subsequent effort and rest periods were defined by force onsets and offsets.

### Maximal force estimate

For all tasks, we measured the maximal force for each hand before starting task performance, following published guidelines [1]. Participants were verbally encouraged to squeeze continuously as hard as they could, until a growing line displayed on a computer screen reached a target. The growing rate was proportional to the force produced to motivate subjects to squeeze hard. Maximal force was set to the average of data points over the last half of the squeezing period exceeding the median. Then subjects were provided a real-time feedback about the force produced on the handgrip, which appeared as a fluid level moving up and down within a thermometer, the maximal force being indicated as a horizontal bar at the top. Subjects were asked to produce a force such that fluid level would reach this horizontal bar and to state whether it truly corresponded to their maximal force. If not, the calibration procedure was repeated.

The procedure was slightly simplified for the Adaptation Tasks and Cost Rating Task: 1) the rate of the growing bar was held constant and not indexed on the participants' exerted force level, 2) the duration during which the participants had to squeeze as hard as they could was fixed to 5 s, and 3) all data points were used for the estimate (and not the last half of recorded levels).

### Behavioral tasks

All tasks were presented on a computer screen, and were programmed with Matlab using Cogent 2000 (Wellcome Department of Imaging Neuroscience, London, UK) for the Implicit and Explicit Tasks, and Psychtoolbox (http://psychtoolbox.org) for the Dissociation Task, Adaptation Tasks, and Cost Rating Task.

#### Adaptation Tasks

The display was quite similar in all adaptation tasks, which included a total of 8 sessions. The exerted force level was always displayed as a fluid moving up a thermometer, the target bar on the top of the thermometer indicating 60% of the participant maximal force. The 60% level was chosen to ensure that effort could be maintained by all subjects and for all the imposed durations. All trials included a first effort (with imposed duration), a rest period, and a second effort (with free duration). The payoff was proportional (with a fixed rate) to the time spent above the target force level during the second effort. The color of the fluid in the thermometer instructed what to do: red for the first effort, blue for rest (with ‘STOP!’ displayed above the thermometer), green for the second effort, which participants initiated either immediately (in Tasks 1 and 2), or at their convenience (in Task 3). When participants stopped squeezing, more precisely at the first force sample under 50% of their maximal force, the color turned to white, instructing that they should rest until the following trial. In all Tasks, ‘PLUS FORT’ (meaning ‘harder’) was displayed above the thermometer during the imposed effort when the force being exerted was under the target level (60%). For Tasks 1 and 2, the color turned to white and the message ‘VOUS AVEZ APPUYE TROP TARD’ (meaning ‘you squeezed too late’) was displayed if the participant initiated the trial too late (more than 1s after the color change). In all three tasks, a flickering dollar symbol was displayed when the force was above the target level, during the second effort whose duration was free (green color), to indicate that money was being accumulated. Both the trial payoff and the cumulated payoff were displayed on screen at the end of each trial.

#### Adaptation Task 1 (variable effort/constant rest/free effort)

Each trial presented the following events successively: imposed effort (at 60% of maximal force), imposed rest (2s), go signal to initiate an effort of free duration (20s allowed), feedback (2s), inter-trial interval (2s). Imposed effort durations were drawn from a set of 36 points regularly spaced between 1 and 10s. The same 36 durations, divided into 4 sessions of 9 trials each, were presented once to the left hand and once to the right hand in the same randomized order. For each session, effort durations were picked up every 4 points in the randomized sequence of 36 values, starting at a sample randomly drawn (without replacement) between 1 and 4. This procedure ensures that over subjects, all sessions have the same average effort duration.

#### Adaptation Task 2 (constant effort, variable rest, free effort)

Each trial presented the following events successively: imposed effort (7s at 60% of maximal force), imposed rest (between 1 and 12.5s), go signal to initiate an effort of free duration (20s allowed), feedback (2s), and no inter-trial interval. Imposed rest durations were defined so as to sample small durations more than long durations. We simulated a mixture of Gaussians (10000 points), with 75% of points drawn from N(3,2), and 25% drawn from N(10,2), where N(m,σ) denotes a Gaussian distribution with mean m and standard deviation σ. This distribution was cut off to retain values higher than 1s and divided into 37 bins. The first 36 bins were then retained for our sampling rest durations to avoid extreme values from the Gaussian distribution. The same 36 durations were presented to the left and right hands in the same randomized order, using the same randomization technique as was implemented for Task 1.

#### Adaptation Task 3 (variable effort, free rest and free effort)

Each trial presented the following events successively: imposed effort (at 60% of maximal force), imposed rest (2s), a signal indicating to the participant that the second effort can be initiated (20s allowed in total for first resting and then exerting effort), feedback (2s), inter-trial interval (2s). Imposed effort durations were 36 points equally spaced between 1s and 10s. The same 36 durations were presented to the left and right hands in the same randomized order, using the same randomization technique as was implemented for Task 1.

#### Cost Rating Task

The task included 7 sessions, using right and left hands alternatively. Each session comprised 21 trials. The design was fully factorial, crossing all factor levels: 3 incentive levels (10c, 20c, 50c), 7 duration levels (equally spaced from 3 to 7s) and 7 difficulty levels (equally spaced from 40 to 60% of the participant maximal force). Each cell was presented only once, as there were 147 cells for 147 trials. The order of presentation was pseudo-randomized such that the different sessions had exactly the same incentive and difficulty level on average, and little variation in mean duration.

Every trial started with baseline (1s), followed by incentive display (1s) and then by the appearance of a thermometer that served as a ‘go’ signal to trigger effort exertion. The fluid level within the thermometer provided online feedback on the force being exerted, with scaling adjusted such that the target bar corresponded to difficulty level (40% to 60% of the maximal force). The thermometer was displayed as long as the participant had to sustain the effort. The imposed duration was applied starting when the target force was reached and not when the thermometer appeared. Exhaustion rating was done just following the effort. ‘Avez-vous épuisé vos ressources?’ (‘Have you exhausted your resources?’) was written on screen, and participants indicated their rating from ‘Pas du tout’ (‘not at all’) to ‘Totalement’ (‘completely’) with a cursor that could be moved with left/right key press. We framed the question in terms of exhaustion instead of the perceived exertion [44] because the rating occurred after (not during) the effort. The rating scale had 50 steps but no visible graduation. Rating and validation (by pressing the space bar) were self-paced. The last screen lasted 1.5 s and summarized the payoff earned in the current trial and the cumulated payoff over all preceding trials. The amount earned during a trial was calculated as the incentive value multiplied by the proportion of the imposed duration spent above the target force level.

#### Effort Allocation Tasks (Implicit, Explicit and Dissociation Tasks)

The Implicit task is described in [10]; we reproduce here the relevant details. Participants performed 8 sessions of 9 trials corresponding to the 9 cells of the factorial design (3 incentive×3 difficulty conditions), which were presented in a random order. Subjects performed 8 sessions in total, switching hands as instructed between sessions to avoid muscular fatigue. After baseline measurement of the pressure at rest, each trial started by revealing the monetary incentive with a coin image (10, 20 or 50 cents) displayed for 1s. Then subjects had 30s to win as much money as possible. They knew that the payoff was proportional to both the incentive and the time spent above the target bar, which was always placed at the same height in the thermometer. The force needed to reach the bar (70, 80 or 90% of subject's maximal force), i.e. trial difficulty, was not indicated to subjects. Subjects only knew that task difficulty would vary across trials. They were provided with online feedback on both the exerted force (with a fluid level moving up and down within a thermometer) and the trial-wise cumulated payoff (with a counter displayed above the thermometer). Each trial ended with a 2s display of the session-wise cumulated payoff.

The only change from the Implicit to the Explicit Task is that the difficulty level was displayed on the right and left of the coin image, as percentages of maximal force (70%, 80% or 90%).

In the Dissociation Task, monetary incentive (10c or 20c), actual difficulty (75% or 85%) and cued difficulty (75% or 85%) were combined into a factorial design comprising 8 cells. Cued difficulty level was indicated on the screen as in the Explicit task but was congruent with the actual difficulty level (actual force needed to reach the target bar) in half the trials only. The experiment was divided into 8 sessions presenting one trial for each of the 8 cells in a random order. The randomization avoided to present identical pair of cues (for incentive and difficulty levels) in two consecutive trials. Apart from the potential mismatch between the cued and actual difficulty levels, the trial settings were identical to those of the Explicit Task.

### Statistical analysis

#### Adaptation Tasks

We first verified that subjects complied with the instructions, meaning that they sustained their effort at the required level (60% of maximal force) and for the imposed duration. As we found no significant deviation from instructed effort, all trials were included in the analysis. Effort and rest durations were analyzed using multiple linear regressions. For Task 1, the dependent variable (second effort duration) was fitted with four regressors: first effort duration, session number, session-wise cumulated effort, and the residual effort initiation delay (i.e., delay between go signal and effort onset, after removing the variance explained by the three other regressors). Significance of parameter estimates was assessed with a random-effect analysis at the group level using a two-sided t-test. For Task 2, the same four regressors were used to explain the dependent variable (second effort duration), except that the manipulated factor was rest duration (not first effort duration). For Task 3, the two dependent variables (rest and second effort durations) were fitted with the same linear model as was done for Task 1, except that there was no initiation delay.

We also analyzed the relationship between imposed and observed durations in Tasks 1 and 2, by fitting linear and saturation models, which we compared using Bayesian model selection (BMS). The linear model was: 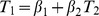. We tested two models for saturation: a bounded linear model:
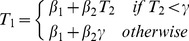
and a model with exponential saturation: 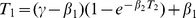, in which β_1_ is the intercept, β_2_ the increase rate and γ the asymptote. The BMS procedure is described in the next section. In principle, the cost-evidence model predicts that recovery during rest should be bounded, such that after a certain time, more rest does not increase the duration of the subsequent effort. For simplicity we assumed a linear dynamics for accumulation and dissipation, which implies that the saturation should manifest as a linear increase followed by a constant plateau. However, we reasoned that white noise in data generation should render this function closer to an exponential saturation. We confirmed this intuition with a simulation, proceeding as follows. 1) We fitted a bounded linear model to individual data and retained the median parameter estimates and the residuals standard deviation for each subject. 2) With these parameter estimates and the durations manipulated experimentally, we simulated 100 sets of noisy effort data per subject, using a white noise of the same magnitude as residuals standard deviation. 3) We fitted both the bounded linear model and the exponential model to these individually simulated data, and calculated their respective log-evidence. 4) We averaged these log-evidences over simulations and performed a group-level BMS, which favored the exponential model with high confidence (chance level is ½, expected frequency ef = 0.75, exceedance probability xp = 0.97). We noticed, as expected, that this exceedance probability decreased when decreasing the noise magnitude. Therefore, we included the exponential model in the BMS performed on our observed data, since it was more likely to capture the saturation effect at the observed noise magnitude.

#### Cost Rating Task vs. Implicit Task

We first submitted the ratings obtained from the Cost Rating Task to a multiple regression analysis, so as to estimate the effect of several factors. The regressors comprised the manipulated factors (incentive, difficulty and duration level) and covariates (a constant per session to capture the mean, a linear trend per session to capture drift over trials, and the initial position of the rating cursor). Two-way interaction terms were also included. Regressors were z-scored over all trials, except trends that were z-scored within their sessions and padded with 0, and constant regressors. The significance of the parameter estimates was assessed with a random-effect analysis at the group level using a two-sided t-test.

To compare with the Effort Allocation (implicit) Task, we modeled cost evidence as follows: 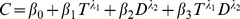, where D and T are difficulty and time (duration). To make the estimation of D and T betas independent of their unit (force versus time), they were divided by their mean value. Setting the λ terms to 1 made the model linear with respect to experimental factors, setting β_3_ to zero made the model purely additive and setting β_1_ and β_2_ to zero made the model purely interactive. All three possibilities (including non-linearities, including additive terms, including interaction) were combined, resulting in a total of 8 models. Note that formally, the linear and non-linear constant models (C = β_0_) are strictly equivalent. In the Cost Rating Task, the dependent variable was subjective rating of cost (sensation of exhaustion), which could be directly regressed against the cost evidence model. In the Effort Allocation (implicit) Task, costs had to be inferred from the behavior. The probability to stop the effort after a given exertion duration was derived from the cumulated distribution of effort duration, for each difficulty level. This probability was regressed against a sigmoid (or logit) function of the modeled cost evidence: 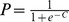. This sigmoid was not parameterized (i.e., C was not transformed with scaling and offset parameters), since this would be redundant with the beta parameters included in the definition of C itself. Apart from the sigmoid transformation, the same procedure was thus applied to the Cost Rating and Effort Allocation Tasks.

A constant elasticity of substitution (CES) model was also fitted to characterize the curvature of cost evidence. The CES model is 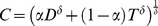, in which α ranges from 0 to 1 and characterizes the equivalence between D and T (or substitution ratio), and δ is strictly positive and characterizes the curvature of this equivalence. We introduced an offset and a scaling factor to the CES model as two additional free parameters, which were independent from the estimation of alpha and delta since D and T had a mean of one. Following the same procedure as in the model comparison, cost evidence was fitted onto subjective rating in the Cost Rating Task, and passed through a logit function to be fitted onto stop probability in the Effort Allocation task.

#### Effort Allocation Tasks (Implicit, Explicit and Dissociation Tasks)

Effort and rest durations were submitted to multiple regression analysis. The regressors comprised the manipulated factors (incentive and difficulty levels for the Implicit and Explicit Task; incentive, cued and actual difficulty levels for the Dissociation Task), temporal factors (the session number, the trial position within a session and the effort or rest position within a trial), and interaction terms (the two-way interactions of manipulated and temporal factors, and the two-way interactions between manipulated factors, which was extended to a third-way interaction between the three manipulated factors in the Dissociation task). All the regressors were z-scored to provide standardized effect size.

The significance of parameter estimates (regression coefficients) was assessed with a random-effect analysis at the group level using a two-sided t-test. Dissociation between cued and actual difficulty in the Dissociation Task was estimated using a two-sided paired t-test on the parameter estimates. For non-parametric t-tests, we estimated the null t-distribution using all possible permutations (n = 2^15^) between the ‘cued’ and ‘actual’ labels, and estimated the probability of t-values more extreme than observed (two-sided test).

### Bayesian model selection

To perform model selection, models were first estimated for each subject following a variational Bayes approach under the Laplace approximation [45], [46], using a toolbox by Jean Daunizeau [47] (available at http://code.google.com/p/mbb-vb-toolbox/). Note that all the models developed here are deterministic: they are meant to provide a mechanistic link from factors of interest (monetary incentive or task difficulty) to observations (effort or rest duration). The aim of model estimation was to find the distribution of free parameters that best fitted the observations, and not to explain their stochasticity. The variational Bayes algorithm not only estimates linear and non-linear models but also calculates their evidence based on a free-energy approximation [45]. The evidence of a model is the probability of observing the data given this model. This probability corresponds to the marginal likelihood, which is the integral over the parameter space of the model likelihood weighted by the prior on free parameters. This probability increases with the likelihood (which measures the accuracy of the fit) and is penalized by the integration over the parameter space (which measures the complexity of the model). The model evidence thus represents a trade-off between accuracy and complexity and can guide model selection [48]. Model selection was performed with a group-level random-effect analysis of the log-evidence obtained for each model and subject, using Gibbs sampling in SPM8 (Statistical Parametric mapping, Wellcome Department of Imaging Neuroscience, London, UK) [48]. This procedure estimates the expected frequency (denoted ef) and the exceedance probability (denoted xp) for each model within a set of models, given the data gathered from all subjects. Expected frequency quantifies the posterior probability, i.e. the probability that the model generated the data for any randomly selected subject. This quantity must be compared to chance level (one over the number of models or families in the search space). Exceedance probability quantifies the belief that the model is more likely than all the other models of the set, or in other words, the confidence in the model having the highest expected frequency [48]. Family-level inference was conducted similarly to model-level inference after defining a partition within the model space as described in [49] and implemented in SPM8.

### Computational models (Implicit, Explicit and Dissociation Tasks)

We first defined a class of models that can a priori produce the results that we intended to explain. These models were then submitted to a Bayesian model selection in order to identify the most plausible model among all the possible models. The model space was defined by simplifying a full model, starting with the Implicit Task. The model is based on accumulation-dissipation processes: cost evidence ramps up during effort to a bound that triggers effort cessation, and ramps down during rest to a bound that triggers effort resumption. As for simplicity the fluctuations were modeled as linear, the effort and rest durations (*Te* and *Tr*) are just the ratios between the amplitude *A* (distance between bounds) and the accumulation or dissipation slope (*Se* and *Sr*). In the full model, the free latent parameters *A*, *Se*, *Sr* can vary across trials around their mean values (α_m_, β_m_, γ_m_), depending linearly on experimental factors: in this case the incentive *I* and the difficulty *D*. The full model is thus:
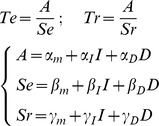
Simpler models can be designed by setting one or more weights to zero. As there are 6 weights (3 latent parameters times 2 experimental factors), all combinations give a total of 2^6^ = 64 models. However, some of these models are not worth considering as they cannot account for the effect that we want to explain. The most extreme case is when all weights are null: such a model cannot produce any of the effect of incentive and difficulty that we observed in the data. After discarding all models with which at least one of the significant results reported in Figure 5 could not be produced (shown with red in Figure 6A), the search space was restricted to 24 models. Note that predicting an effect which was not significant in our data was not a criterion for rejection.

To illustrate the logic of model selection, we can take the case of the opposite effects of incentives on effort and rest duration (Te and Tr). This behavioral pattern cannot be accounted for by models which would have no incentive effect at all or an effect on one latent parameter only (A, Se or Sr). These models can therefore be excluded from the model space retained for Bayesian comparison. On the contrary, modulations of both A and Se (#1), or both A and Sr (#2), or all three parameters (#3) can a priori produce the observed incentive effects on Te and Tr; these models should therefore be compared. Model #1 predicts that incentive effect on Tr should be linear (because it only affects the numerator A) and the effect on Te non-linear (because it also affects the denominator Se). Model #2 generates the opposite predictions. The data can thus disambiguate between these models, depending on which effect is non-linear. Model #1 and #2 are special cases of model #3 which is more complex and thereby, more likely to provide a better fit. Yet model #3 will be preferred over model #1 and #2 only if the improvement of fit surpasses the inflation of complexity in the calculation of model evidence. Note that this example is a simplification of our model comparison, as all factors (not just incentives) should be considered at the same time, which also makes predictions on the pattern of interaction between factors. However, detailing the specific predictions of all the models included in the Bayesian comparison would require much more length than that allowed in a research paper.

The same approach was applied for defining linear models of the Explicit Task, leading to a search space of 16 models (Figure 6C). For the Dissociation Task, another modulator was included as there were two types of difficulty (cued and actual). The full model has therefore 9 weights, which gives 2^9^ = 512 possible models, which were reduced to 144 models after rejection of irrelevant models (Figure 6B).
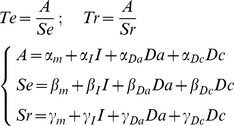
Since these models provided poor fit and unclear evidence in favor of a particular model, we also tested a class of hyperbolic models for the Explicit and Dissociation Tasks. As opposed to the linear formulation, the discount of incentive by cued difficulty was assumed to be hyperbolic, as in some economic models of temporal discounting. The full hyperbolic model is:
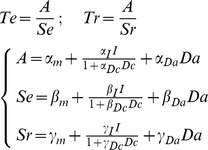
The *D* term that denoted difficulty in the first linear model has been decomposed into *Da* and *Dc*, denoting actual and cued difficulty in the Dissociation Task. Note that in the Explicit Task, *De* and *Dc* have exactly the same values. The model can nonetheless be estimated unambiguously in this task since the effect of *Da* is linear whereas that of *Dc* is hyperbolic. Also note that with hyperbolic formulation, there are dependencies between weights since a null numerator prevents the denominator from impacting the model fit. Thus we discarded models with a null numerator and a non-null weight at the denominator (this is shown with red in Figure 6D). After discarding the models that were not able to produce all the significant results shown in Figure 5, the search space was eventually restricted to 78 models for the Explicit and Dissociation Tasks.
